# Preparing healthcare leaders of the digital age with an integrative artificial intelligence curriculum: a pilot study

**DOI:** 10.1080/10872981.2024.2315684

**Published:** 2024-02-13

**Authors:** Soo Hwan Park, Roshini Pinto-Powell, Thomas Thesen, Alexander Lindqwister, Joshua Levy, Rachael Chacko, Devina Gonzalez, Connor Bridges, Adam Schwendt, Travis Byrum, Justin Fong, Shahin Shasavari, Saeed Hassanpour

**Affiliations:** a Geisel School of Medicine at Dartmouth, Hanover, NH, USA; b Department of Radiology, Stanford Medicine, Palo Alto, CA, USA

**Keywords:** Artificial intelligence, machine learning, data science, undergraduate medical education, preclinical training

## Abstract

Artificial intelligence (AI) is rapidly being introduced into the clinical workflow of many specialties. Despite the need to train physicians who understand the utility and implications of AI and mitigate a growing skills gap, no established consensus exists on how to best introduce AI concepts to medical students during preclinical training. This study examined the effectiveness of a pilot Digital Health Scholars (DHS) non-credit enrichment elective that paralleled the Dartmouth Geisel School of Medicine’s first-year preclinical curriculum with a focus on introducing AI algorithms and their applications in the concurrently occurring systems-blocks. From September 2022 to March 2023, ten self-selected first-year students enrolled in the elective curriculum run in parallel with four existing curricular blocks (Immunology, Hematology, Cardiology, and Pulmonology). Each DHS block consisted of a journal club, a live-coding demonstration, and an integration session led by a researcher in that field. Students’ confidence in explaining the content objectives (high-level knowledge, implications, and limitations of AI) was measured before and after each block and compared using Mann-Whitney *U* tests. Students reported significant increases in confidence in describing the content objectives after all four blocks (Immunology: *U* = 4.5, *p* = 0.030; Hematology: *U* = 1.0, *p* = 0.009; Cardiology: *U* = 4.0, *p* = 0.019; Pulmonology: *U* = 4.0, *p* = 0.030) as well as an average overall satisfaction level of 4.29/5 in rating the curriculum content. Our study demonstrates that a digital health enrichment elective that runs in parallel to an institution’s preclinical curriculum and embeds AI concepts into relevant clinical topics can enhance students’ confidence in describing the content objectives that pertain to high-level algorithmic understanding, implications, and limitations of the studied models. Building on this elective curricular design, further studies with a larger enrollment can help determine the most effective approach in preparing future physicians for the AI-enhanced clinical workflow.

## Introduction

In the era of big biomedical data and artificial intelligence (AI), the recent emergence of flexible natural language processing models, including ChatGPT, has been considered an inflection point in AI-driven healthcare and biomedical research [1,2]. Despite the constantly changing landscape of clinical practice, there is a pervasive lack of confidence in and unfamiliarity with AI among physicians [3–5]. While the ethical and legal dilemma associated with such technology remain uncertain, implications of AI models like ChatGPT in the clinical workflow are limitless, ranging from summarizing the electronic health records and alleviating the burden of preauthorization processes to improving the interpretability of computer-aided diagnoses and empathetically responding to routine patient queries online [2,6]. As these technologies continue to evolve at a much faster pace than their regulators, it is imperative to equip future physicians with the necessary AI knowledge and confidence to not only communicate with patients regarding the appropriate utility of the present technology but also direct the implementation of future applications as they apply to patient care [1,7,8].

Despite the increasing recognition of the benefits of digital health education for future physicians, no clear consensus exists on how to most effectively deliver the AI curriculum as part of undergraduate medical education (UME) [9–11]. Similar efforts to introduce clinically relevant adjunctive topics, such as medical ethics and nutrition, into the traditional UME suggest that longitudinal integration of new concepts into ongoing preclinical curriculum may better help medical students apply the newly learned concepts [12,13]. However, requiring all medical students to engage in a digital health curriculum in addition to the robust volume of preclinical material may represent a significant added burden for students with alternative interests or those wanting to focus on satisfying other core competencies needed for residency [14,15]. Hence, an optional digital health elective that offers an AI curriculum that integrates data science concepts by paralleling the ongoing preclinical courses may represent a potential means to prepare a cohort of interested physicians as leaders of the digital health workforce.

Our pilot study aimed to evaluate the effectiveness of a medical school digital health non-credit enrichment elective that focused on longitudinal integration of increasingly complex AI concepts into the preclinical education. Specifically, we aimed to assess whether medical students who receive AI education in the pilot digital health curriculum demonstrate an improvement in their self-reported confidence in explaining the elective’s learning objectives (high-level knowledge, implications, and limitations of AI). Despite the increasing application of AI in medicine, the general patient population still carries a distrust in AI-informed care [16]; with medical students’ enhanced confidence in explaining AI capabilities in medicine, future physicians may be able to improve communication and ameliorate this distrust. The results of this study may provide insights into how to best integrate AI education into medical school curricula and speak to the effectiveness of paralleled longitudinal integration as a viable strategy to prepare medical students for the rapidly changing digital healthcare landscape.

## Materials and methods

The Digital Health Scholars (DHS) enrichment elective curriculum (September 2022 - March 2023) was designed for first-year (M1) medical students at the Dartmouth Geisel School of Medicine with the aim of integrating AI concepts and their biomedical research applications into their preclinical curriculum by paralleling their classroom studies. M1 students were selected, as the institution offers enrichment electives only in the preclinical years, and as second-year (M2) students were likely to be more focused on Step 1 preparation than to engage in an enrichment elective. After reviewing each of the first four systems blocks of the M1 preclinical curriculum (Immunology, Hematology, Cardiology, Pulmonology) and filtering for clinically relevant material, we chose a topically related research paper for each subject. Three learning modules were then built around each selected paper: 1) a foundational journal club that reviewed the data science concept used in the study (i.e., convolutional neural network), 2) a demonstration of the methodology in a live-coding session on JupyterNotebook using Python programming language, and 3) an integration session led by a speaker who was part of the AI study (i.e., a study that predicts lung cancer mutation status using convolutional neural network on lung histology sections) that led to the paper. The use of journal clubs has been proven to be effective in introducing AI concepts to medical professionals from various statistical backgrounds [17]. The three modules served to satisfy the specific learning objectives for each block, which pertained to a high-level understanding of the chosen algorithm, its limitations, and its implications. Additionally, each DHS block also aimed to continuously reinforce core principles that underpin every machine learning model introduced: data preprocessing, model construction and validation, and model translatability. Research articles and learning objectives pertaining to each DHS block have been summarized in Table 1.

**Table 1.** Research articles of focus and four content objectives for each 2022–2023 digital health scholars (DHS) block. 5-point likert scales were used to assess the confidence in each of these objectives before each journal club and after each integration session.

DHS Blocks	Author(s), year, and reference	Objectives
Immunology	Natarajan et al., 2022 [18]	Describe the high-level mechanism of multivariate linear regression in solving linear regression problems.
		Explain how the beta-coefficients relate to the features in modeling.
		Explain the limitations of this approach in predicting neutralization activity.
		Describe the implications of univariate/multivariate linear regression models in immunology.
Hematology	Dunn et al., 2021 [19]	Describe the high-level mechanism of Decision Trees.
		Describe the high-level mechanism of Random Forest.
		Describe the utility of wearables in measuring vitals, compared to vitals measured in clinics.
		Describe the utility of wearables in making personalized predictions of lab values.
Cardiology	Brown et al., 2013 [20]	Describe the difference between linear regression and logistic regression.
		Describe the high-level mechanism of univariate and multivariate logistic regression in solving classification tasks
		Describe the implications of univariate/multivariate logistic regression models in risk factor identification.
		Understand the idea of goodness-of-fit statistics.
Pulmonology	Tomita et al., 2022 [21]	Describe the indications for using neural networks.
		Describe the high-level mechanism of Convolutional Neural Networks in solving classification tasks
		Describe the implications of neural networks in predicting mutation/tumor types.
		Understand the limitations regarding models’ generalizability.

**Figure 1.**
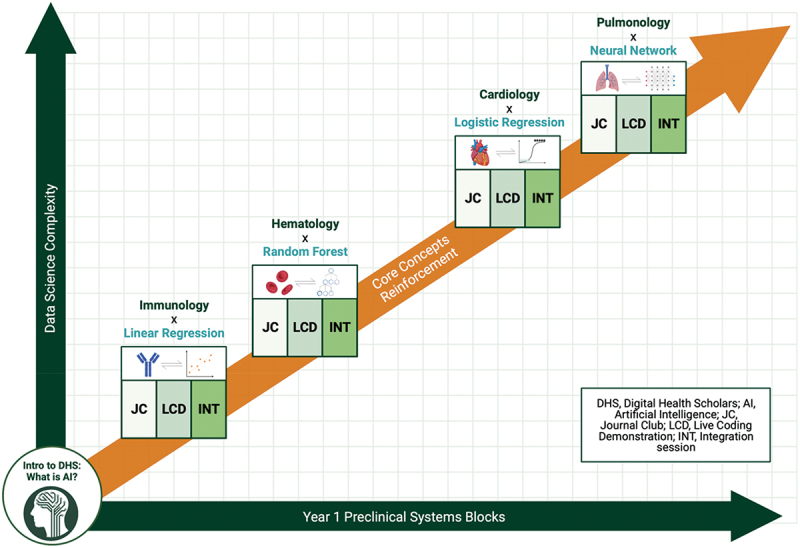
Overview of the 2022–2023 digital health scholars (DHS) program at the Dartmouth Geisel school of medicine that parallels the first-year preclinical curriculum. Each of the four DHS blocks consisted of three modules: a journal club and a coding demonstration that were together held in one session, followed by an integrative lecture-discussion.

We invited M1 students interested in the elective to write a short essay describing their motivation to study digital applications in healthcare to demonstrate their willingness to engage in the curriculum. For the initial pilot curriculum, a total of ten students were enrolled in the DHS curriculum designed by two faculty members at our institute’s quantitative biomedical science department and five M2 students. Of the ten enrolled students, seven indicated that they knew how to use a programming language and had computational research experience. Before the start of the four DHS blocks, an introductory lecture was held to describe the history and current state of AI in medicine. Subsequently, every DHS block contained an opening session that includes a journal club and a coding demonstration, followed by a closing integration session. Although in-person attendance was encouraged whenever possible, every session was hybrid with an online attendance option to accommodate for schedule and location conflicts. As a medical education study, this study was granted exemption from requiring ethics approval by Dartmouth College’s Committee for Protection of Human Rights on 13 June 2022 (#STUDY00032539). A summarized diagram of the DHS curriculum is shown in Figure 1.

### Journal club and coding demonstration

Every journal club was one hour long in length and was led by an M2 medical student DHS curriculum creator approximately one week before its linked integration session in order to provide the enrolled M1 students with the foundational data science knowledge and ease their understanding of its research application in a medical field. The enrolled M1 students were provided with and encouraged to read the research article of focus in advance of each journal club. The M2 students who led the sessions either possessed a bachelor’s degree or higher that utilizes advanced statistics/data science (e.g., computer science, statistics, engineering) or were proficient in one or more programming languages. These student leaders began creating their session content during their M1 summer, and their readiness to lead these sessions were assessed by a professor of biomedical science and other student leaders reviewing the journal club material.

**Figure 2.**
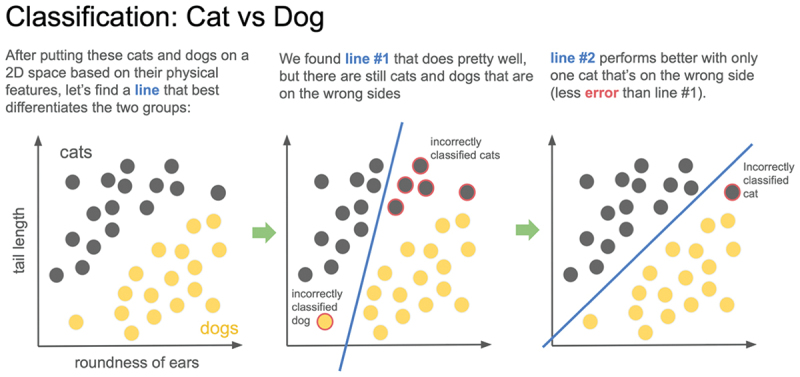
Sample lecture slide from digital health scholars journal club 2: hematology and random forest (section on review of different types of classification).

The content of each journal club was determined by the integration speaker’s research, and each session focused on providing a high-level overview of an algorithm with real-life examples, rather than explaining the underlying mathematical concepts. Jargon was limited as much as possible, and any computational concepts that could overwhelm inexperienced medical students were explained in a simplified fashion (Figure 2) [14]. After introducing the different foundational forms of regression with applications in immunology and classification with applications in hematology and cardiology, the curriculum culminated with the introduction of deep learning algorithms, namely convolutional neural networks, a more advanced form of machine learning that utilizes a significantly higher number of features.

At the end of each journal club, a live-coding demonstration was held, time permitting, using public datasets to illustrate how an AI model is implemented, including information on model training and validation. Here, in addition to active group discussions, students were given time and encouraged to practically engage with the materials by running and editing the pre-written JupyterNotebook code. We believed that having the enrolled students follow along the core principles within the lines of code to be an appropriate way for them to gain exposure to a computing language and confidence in communicating with technology developers and supervising AI tools in the future [22,23], while maintaining their interest in AI. All journal club and live-coding materials were co-created with a professor of biomedical data science.

### Integration session

After students were introduced to the data science concepts used in the research pertaining to preclinical concepts from each block through a journal club, they engaged in a more formal lecture-discussion led by the researcher who performed the work. The aim of each integration session was to solidify the students’ data science knowledge and expand upon the implications and limitations of the AI model in the particular field. For example, students were encouraged to think critically about what it meant to use only an internal institutional dataset to train a model and the implications for the generalizability of such a model. Additionally, each session included a discussion on the potential for translational applications of the respective technologies for existing clinical workflows.

### Assessment

For each of the four DHS blocks, the students enrolled in the curriculum were administered a pre-survey at the start of the journal club and a post-survey at the end of the integration session. Both surveys administered on the Qualtrics platform (Qualtrics, Provo, Utah) consisted of a student’s rating of their own confidence in describing each of the four learning objectives of the DHS block on a 5-point Likert scale. Furthermore, each post-survey also assessed the students’ satisfaction with the paper selection, depth of the content, and how well the learned material applied to the preclinical block. Student attendance level was gathered for each session, and differences in the students’ confidence in explaining the learning objectives between before and after each DHS block were assessed using Mann-Whitney *U* tests. Non-parametric testing was used due to the use of ordinal variables and our sample size. Descriptive statistics were gathered from the satisfaction level of the post-surveys.

## Results

### Confidence in describing learning objectives

**Figure 3.**
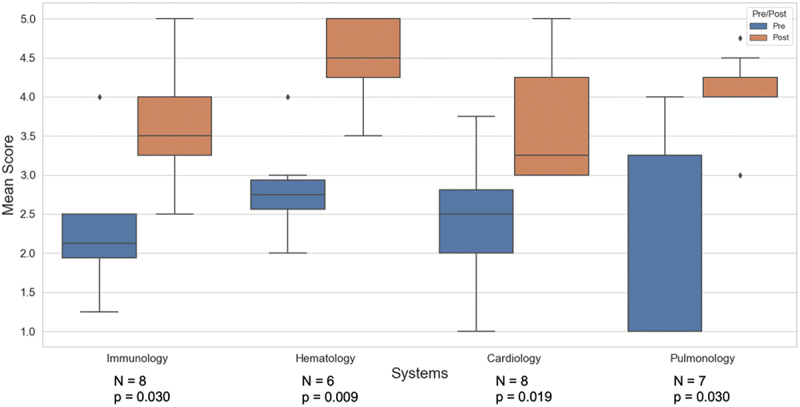
Box plot of survey results from first-year medical students enrolled in the elective describing the degree of confidence in explaining the content objectives of the block (1 = not confident, 5 = extremely confident) before and after each DHS block. Results of the associated Mann-Whitney *U* tests comparing pre- and post-data as well as attendance at each DHS session are shown.

Results of the Mann-Whitney *U* tests indicated significant increases in students’ self-reported confidence in describing the content objectives after all four blocks (Immunology: *U* = 4.5, *p* = 0.030; Hematology: *U* = 1.0, *p* = 0.009; Cardiology: *U* = 4.0, *p* = 0.019; Pulmonology: *U* = 4.0, *p* = 0.030). These levels of confidence for each pre/post-surveys as well as attendance for each session are shown in Figure 3.

### Satisfaction of DHS blocks

Students’ average levels of satisfaction in researcher paper selection, depth of the content, and the integration component of individual blocks ranged from 3.67 to 5/5. A summary of these measures is presented in Table 2.

**Table 2.** Average measures of satisfaction for each digital health scholars block reported by first-year medical students enrolled in the elective.

Satisfaction	Immunology	Hematology	Cardiology	Pulmonology
Comfort in explaining the journal article that was of focus	3.67 (0.52)	4 (1.00)	3.88 (0.83)	3.86 (0.69)
Data Science Concept	3.67 (0.82)	3.80 (0.84)	4.13 (0.64)	4.14 (0.38)
Appropriateness of the paper	4.33 (0.52)	4.6 (0.55)	4.50 (0.53)	4.57 (0.53)
Depth of the content	4.17 (0.41)	4.8 (0.45)	4.50 (0.53)	4.71 (0.49)
Integration of the Journal Club with the speaker	4.17 (0.41)	5 (0.00)	4.50 (0.76)	4.71 (0.49)

## Discussion

The DHS elective was well received by participating students, with an overall satisfaction score of 4.29 out of 5. At the end of each DHS block, students reported a high level of satisfaction (≥4.00) with paper appropriateness, depth of content, and course integration. These findings suggest that students maintained a high degree of interest in data science and AI-focused education. Additionally, student self-perceived confidence significantly improved as illustrated by the pre-post surveys for each of the four DHS blocks, further supporting that the DHS elective may be an effective model in enhancing preclinical medical student confidence in explaining data science and AI content objectives in the domains of high-level algorithmic understanding, implications of use, and limitations of technology.

Prior efforts to teach AI in medical school have been heterogeneous in their educational methods yet have noted both a lack of faculty familiarity and limited curricular time as significant challenges of implementation into preclinical curricula [9,14,24,25]. Our DHS elective attempted to circumvent these limitations by focusing on high-level understanding of the AI algorithms and embedding tailored individual sessions into preclinical organ system ‘blocks’ [9,22].

Paralleling the preclinical curriculum, we selected recent research articles that not only pertained to a specific organ system ‘block’ but also used an algorithm with a specific level of difficulty. We are aware that in medical education that multiple exposures to the same topic in both familiar and unfamiliar contexts over time can improve retention and understanding [12]. Following this established longitudinal framework, we continued to reinforce core data science concepts while introducing AI models in increasing complexity [17,26]. Our students’ satisfaction with the enrichment elective and their increased confidence in explaining content objectives together suggest that having students engage in discussions of novel AI research on a schedule aligned with the concurrent organ system ‘blocks’ is a viable model for teaching the necessary AI topics within an already overcrowded UME curriculum. It must be noted, however, that the participants were *self-selected* students interested in AI-informed care. Hence, the reported outcomes may have been higher than if the entire medical student cohort was taught the same curriculum. Additionally, it is also noteworthy that despite seven out of ten M1 students having prior programming/computational biomedical research experience, the students’ satisfaction in data science concept delivery was lower than those in other domains, warranting a careful balance of technical and applicational loads in the curriculum, especially if the course were to be offered to all M1 students.

One limitation of our study includes heterogeneity of student attendance. Due to the nature of a non-credit enrichment elective model, medical students may have prioritized core preclinical competencies that are required for the medical licensing exams over session attendance [14]. Full engagement in these electives will likely remain limited until board examinations start placing importance on digital health and AI in UME [14,22]. Building on this pilot design, future studies may aim for a higher enrollment of students to help identify the most effective teaching model. Moreover, the enrichment elective as structured was short individual sessions focusing on high-level understanding of the algorithms; for such reason, it was only able to demonstrate the programming process and did not teach the students how to code. With limited time in medical school, teaching those with varied statistical and computational backgrounds to code poses significant implementation challenges [26,27]. Hence, future iterations may focus more on practical learning by creating teams with programmers and data scientists to implement hands-on projects that require AI model building and testing. In addition to assessing students’ skill proficiency through pre-post multiple choice mastery questions, tasking the enrolled students, as part of case-based learning sessions, to explain the concepts in their own words may represent an innovative way to approximate their knowledge, confidence, and AI-related communication skills. It will also be important to administer free-text commentary surveys to explore other avenues for improvement and follow up with the enrolled students to examine whether their confidence in explaining the content objectives remains high even after they begin their clinical training. Other options for expanding the DHS elective may include increased emphasis on the utility of AI in clinical decision making, ethical and legal implications of the use of AI in healthcare, and the responsibility of future physicians, as primary users and evaluators of such technology [28,29]. As the role of AI continues to grow in healthcare, it will also be critical to highlight the limits of AI and emphasize the importance of interpersonal and communication skills for clinicians [28,30].

This study was a response to a growing need to train future physicians who are equipped with foundational AI and data science knowledge and their applications. Confidence in explaining such concepts will be necessary for integration of new and emerging technology into the healthcare space and improved communication with patients regarding their AI-informed care. Our DHS enrichment elective may represent an effective model for integrating AI and data science into the preclinical UME.

## Supplementary Material

DHS_TitlePage.docx
