# Supplementary material for: Preparing healthcare leaders of the digital age with an integrative artificial intelligence curriculum: a pilot study
Source: Med Educ Online. 2024 Feb 13;29(1):2315684. doi: 10.1080/10872981.2024.2315684 (PMC10868429; doi:10.1080/10872981.2024.2315684)
Supplement: DHS_TitlePage.docx [file ZMEO_A_2315684_SM7330.docx]

**Preparing Healthcare Leaders of the Digital Age with an Integrative Artificial Intelligence Curriculum: A Pilot Study**

Soo Hwan Park^a,*^, Roshini Pinto-Powell^a^, Thomas Thesen^a^, Alexander Lindqwister^b^, Joshua Levy^a^, Rachael Chacko^a^, Devina Gonzalez^a^, Connor Bridges^a^, Adam Schwendt^a^, Travis Byrum^a^, Justin Fong^a^, Shahin Shasavari^a^, & Saeed Hassanpour^a^

^a^Geisel School of Medicine at Dartmouth, Hanover, NH, United States

^b^Department of Radiology, Stanford Medicine, Palo Alto, CA, United States

**^*^Corresponding Author**

Soo Hwan Park

1 Rope Ferry Rd

Hanover, NH 03755

Tel.: +1-802-698-2535

E-mail address: [soo.hwan.park.med@dartmouth.edu](mailto:soo.hwan.park.med@dartmouth.edu)

**Word Count:** 2561

**Acknowledgements**

The authors would like to thank the invited speakers, Shiwei Xu, Jessilyn Dunn PhD, Jeremiah Brown PhD, and Liesbeth Hondelink MD, for their contributions to reviewing the data science concepts and leading the integrative discussions. The authors have not declared a specific grant for this research from any funding agency in the public, commercial or not-for-profit sectors.

**Disclosure of Interest:** The authors report there are no competing interests to declare.

**Funding:** The authors have not declared a specific grant for this research from any funding agency in the public, commercial or not-for-profit sectors.

**Ethics approval:** This is a medical education study. Exemption from ethics approval was granted by Dartmouth College’s Committee for Protection of Human Rights on June 13, 2022 (#STUDY00032539).
